# Comparing the Molecular Pharmacological Properties of Existing β‐Blockers to Determine the Theoretically Most “Ideal” Anti‐Cancer β‐Blocker

**DOI:** 10.1002/prp2.70214

**Published:** 2026-01-03

**Authors:** Jillian G. Baker

**Affiliations:** ^1^ Cell Signalling, COMPARE, School of Life Sciences, C Floor Medical School Queen's Medical Centre, University of Nottingham Nottingham UK; ^2^ Respiratory Medicine, Sherwood Forest Hospitals NHS Trust, King's Mill Hospital Nottinghamshire UK; ^3^ Respiratory Medicine, Nottingham University NHS Trust Queen's Medical Centre Nottingham UK

## Abstract

There is increasing evidence, from cellular, animal and human epidemiological studies, linking β‐blockers with reductions in cancer growth and metastasis. Propranolol is the most investigated β‐blocker for cancer; although as many different off‐patent β‐blockers exist, there is little commercial incentive to drive comparative clinical trials. To minimize any chance of endogenous β‐agonist driven cancer growth or metastasis, theoretically, the “ideal” anti‐cancer β‐blocker would have high affinity, no partial agonism, and long duration of action at β2‐adrenoceptors (and for some cancers, additionally at β1 or β3‐AR). Using CHO cells stably expressing the wildtype and polymorphic variants of the human β1 and β1‐adrenoceptors, this study assessed 35 β‐blockers for the affinity and duration of binding (using ^3^H‐CGP12177 whole cell binding) and intrinsic efficacy (CRE‐gene transcription). Despite high affinity, some β‐blockers had a short binding duration (e.g., alprenolol, bupranolol, levobunolol, nadolol and oxprenolol). Other compounds had substantial partial agonism (e.g., cyanopindolol, bucindolol, pindolol, pronethalol and xamoterol) and other compounds had a biphasic washout (e.g., bucindolol, timolol, carpindolol, and CGP12177) for reasons unknown. Considering all 3 factors, carazolol and ICI118551 may be more “ideal” than propranolol; however, carvedilol, with higher affinity and substantially longer duration of β2 (and β1) receptor binding than propranolol whilst maintaining low partial agonism, may be the most theoretically optimal. Furthermore, it is already widely used in cardiovascular medicine as an off‐patent tablet. Thus, carvedilol may have more optimal molecular pharmacological characteristics for an “anti‐cancer” β‐blocker than propranolol and could enter prospective comparative clinical trials without needing any further clinical workup.

## 1 Introduction

β‐antagonists (β‐blockers), including propranolol, were developed in the 1960s to block endogenous catecholamine activation of cardiovascular β‐adrenoceptors (β‐AR), reducing cardiac output and improving angina and hypertension [1]. β‐antagonists are widely used for heart failure, arrhythmias, ischaemic heart disease, thyrotoxicosis, migraine, anxiety, portal hypertension, cirrhosis and variceal bleeding, hypertension, and glaucoma [2]. In 2008, propranolol, given to two children with cardiovascular instability from infantile haemangiomas, was found serendipitously to cause tumor regression [3]. Propranolol rapidly became the first‐line treatment for this benign, but sometimes troublesome, vascular tumor [4]. Cancer studies suggest increasing evidence that β‐blockers may improve outcomes (including breast, colon, melanoma, ovarian, prostate, liver, esophagus, lung, stomach, head and neck cancers, multiple myeloma, and angiosarcoma) through a variety of catecholamine‐driven pathways including modulating metabolism, proliferation, invasion and apoptosis of cancer cells, and affecting tumor vasculature, inflammation, and immunity (see Ref. [5, 6, 7, 8, 9, 10, 11, 12, 13, 14, 15] for detailed reviews). β2‐ARs are the most commonly implicated β‐AR (e.g., Ref. [5, 16] and references above and below); however, some cancers do express β1‐ARs and β3‐ARs for example, [8, 17, 18, 19].

In laboratory studies, catecholamines increased growth and/or migration in cancer cell lines that were reduced by β‐blockers (e.g., propranolol [20, 21, 22], propranolol and the β2‐selective antagonist ICI118551 [17], carvedilol [23]). In animals, increased catecholamines (physiologically induced or directly administered) increased cancer growth [6, 18, 23, 24, 25, 26, 27, 28, 29], which was reduced by β‐blockers (propranolol [24, 25, 26, 27, 28, 29], propranolol and ICI118551 [18], carvedilol [23]). Propranolol and ICI118551 also reduced cancer growth and increased survival in animal studies of cancers induced by other mechanisms, demonstrating that inhibition of endogenous catecholamines was beneficial [30, 31, 32].

Retrospective epidemiological studies of cancer patients prescribed β‐blockers for non‐cancer indications are linked with improved cancer outcomes [33, 34, 35, 36, 37, 38, 39, 40, 41, 42], despite β‐blockers being mainly aimed at cardiac β1‐blockade and the understandable complications of defining β‐blocker “exposure” (which may be a single β‐blocker prescription during the entire study). A different study examined β‐blocker usage (carvedilol) in a large cancer‐free population and found the cumulative 12‐year incidence of subsequent cancer development was 26% lower for carvedilol users than non‐users [43].

Prospective β‐blocker cancer studies are few. A prospective study of patients offered propranolol at melanoma diagnosis showed a reduction in disease progression, all‐cause mortality, and melanoma death at 3 years (overall 80% reduction in risk of disease progression [44]). However, this is not a universal finding. Data gathered prospectively in ovarian cancer patients concomitantly using β‐blockers found “β1‐selective” β‐blocker‐users (defined as β‐blocker use documented once or more over the 4 years), but not “non‐selective” β‐blocker‐users, had higher mortality than non‐users (although β‐blocker‐users were older, more obese with more co‐morbidities [45]). See Ref. [10, 13, 46] for reviews including other neutral or negative retrospective epidemiological studies.

As there are many off‐patent, widely used, affordable, oral β‐blockers, repurposing an existing β‐blocker to improve cancer outcomes would take minimal effort and cost, and be immediately available at cancer diagnosis, regardless of other planned treatments [47]. However, existing β‐blockers have different molecular pharmacological profiles that affect clinical cardiovascular outcomes. For example, several “neutral antagonist” β‐blockers reduced mortality during the acute MI period (timolol by 39% [48], metoprolol by 36% [49], propranolol by 26% [50]), whilst β‐blockers with moderate partial agonism (known clinically as intrinsic sympathomimetic activity, ISA) were not beneficial (e.g., oxprenolol and pindolol [51, 52, 53]). In heart failure patients, “neutral antagonist” compounds demonstrated a 34%–35% reduction in all‐cause mortality (metoprolol [54], bisoprolol [55], carvedilol [56]), whereas ligands with substantial partial agonism were either not beneficial (bucindolol [57]) or actually increased mortality (xamoterol [58]). Different molecular pharmacological profiles are likely to affect cancer outcomes too, and identifying the best “anti‐cancer” profile may be important for best clinical outcome. Evidence suggests that β‐blockers are not equally effective in cancer. Epidemiology studies suggest “non‐selective” β‐blockers appear better than “β1‐selective” agents (e.g., Ref. [34, 40]), and migration of colon and prostate cells was reduced more by propranolol and ICI118551 than by atenolol [17, 20].

Studies suggest that endogenous catecholamine blockade, particularly at β2‐AR, is important for anti‐cancer effects. Therefore, in theory at least, to minimize any potential endogenous catecholamine‐driven cancer growth or metastasis, logically, the ideal molecular pharmacological properties for an anti‐cancer β‐blocker would be (1) high β2‐AR affinity (β1‐AR and β3‐AR antagonism may be helpful in some cancers), (2) long duration of β2‐AR binding (to fully occupy all β2‐AR all of the time and prevent any adrenaline/noradrenaline‐driven cancer growth) and (3) no partial agonism. Propranolol is the most investigated β‐blocker in cancer studies; however, it may not be the “best” β‐blocker for cancer. This study therefore examined these three molecular pharmacological properties (affinity, duration of binding and efficacy) for 35 β‐blockers for the human β2 and β1‐AR, ranked compounds for each property, and compared the overall properties with those of propranolol to determine which would theoretically have the best overall molecular pharmacological profile as a potential anti‐cancer β‐blocker.

## 2 Materials and Methods

### 2.1 Materials

^3^H‐CGP12177 was from Amersham International (Buckinghamshire, UK) and Microscint 20 and Ultima Gold XR scintillation fluid from PerkinElmer (Shelton, CT, USA). L‐glutamine (G7513), Dulbecco's modified Eagle's medium nutrient mix F12 (DMEM/F12: D6421), fetal calf serum (F7524), diethanolamine (D8885), and p‐NPP (71768) were from Sigma Chemicals (Poole, Dorset, UK). NDD825 was from [59]. A full list of the ligands used is in Supporting Information together with product number and supplier (Table S1).

### 2.2 Cell Culture

Cells used in this study were all CHO cells (CHO‐K1 RIDD: CVCL_0214) and were grown in Dulbecco's modified Eagle's medium nutrient mix F12 (DMEM/F12) containing 10% foetal calf serum and 2 mM L‐glutamine in a 37°C humidified 5% CO_2_: 95% air atmosphere. All cell lines contain one stably transfected human β‐adrenoceptor and a CRE‐SPAP reporter gene. Cell lines used and original descriptions are: wild‐type β2 = CHO‐β2 [60], wild‐type β1 = CHO‐β1 [61], cell lines expressing the 4 naturally occurring β2‐polymorphisms CHO‐β2‐gly16, CHO‐β2‐gln27, CHO‐β2‐met34, CHO‐β2‐ile164 [62] and cell lines expressing 2 naturally occurring β1‐polymorphisms CHO‐β1‐gly49 and CHO‐β1‐arg389 [63]. No antibiotics were used throughout the study and cell lines were intermittently screened throughout the study for mycoplasma and were negative.

### 2.3 ^3^H‐CGP12177 Whole Cell Binding—Determination of ^3^H‐CGP12177 Affinity

Cells were grown to confluence in white‐sided, tissue culture treated 96‐well view plates. The *K*_D_ for ^3^H‐CGP12177 was determined from saturation experiments whereby media was removed from all wells, 100 μL serum free media (SFM = DMEM/F12 containing and 2 mM L‐glutamine) or 100 μL SFM containing 20 μM propranolol (to define non‐specific binding) added to the wells immediately followed by 100 μL ^3^H‐CGP12177 in SFM (1:2 dilution in well, quadruplicate wells/plate) in increasing concentrations from 0.007 nM to 30.3 nM. After 2 h at 37°C plates were washed with 2 × 200 μL 4°C PBS, 100 μL Microscint 20 added to each well, and after several hours in the dark, counted on a Topcount for 2 min per well. The concentrations for each dilution of ^3^H‐CGP12177 used were also determined in triplicate for each experiment using a TriCarb scintillation counter (3 min count per vial).

### 2.4 ^3^H‐CGP12177 Whole Cell Binding—Determination of β‐Antagonist Affinity

The affinity (*K*_D_) of β‐antagonists was determined from competition experiments as in [64, 65]. Media was removed from confluent cells, 100 μL competing ligand (in SFM at twice final concentration, triplicate wells/plate) added to each well immediately followed by 100 μL fixed concentration of ^3^H‐CGP12177 in SFM to give final ^3^H‐CGP12177 concentrations in the range of 0.43–1.14 nM. After 2 h at 37°C the cells were washed with 2 × 200 μL 4°C PBS, 100 μL Microscint 20 added to each well, and after several hours in the dark, counted on a Topcount for 2 min per well. These formed the control plates for the assessment of duration of binding.

### 2.5 ^3^H‐CGP12177 Whole Cell Binding—Determination of β‐Antagonist Duration of Binding

A relative measure of duration of receptor binding was achieved as described in [65]. In a parallel plate to the control plate, cells were incubated with 100 μL of the β‐antagonist + 100 μL SFM for 2 h (known as the wash plate = competing ligand alone). After 2 h this wash plate was washed with 2 × 200 μL warm SFM. 100 μL ^3^H‐CGP12177 + 100 μL SFM was added to the wells = ^3^H‐CGP12177 alone (except non‐specific binding wells when propranolol was re‐added) and incubated for 2 h. After this, the plates were washed with 2 × 200 μL 4°C PBS, 100 μL Microscint 20 added to each well, and after several hours in the dark, counted on a Topcount for 2 min per well. Total and non‐specific binding were determined in all control and wash plates, and as the wash plates had more washes than the control plate (with more potential for cell loss), the data were normalized to the total and non‐specific binding values measured in each plate.

### 2.6 CRE‐SPAP Gene Transcription

CRE‐SPAP production was measured as first described in [66]. Confluent cells in clear‐sided, tissue culture treated 96‐well plates were serum‐starved (media removed and replaced by 100 μL SFM per well) for 24 h before experimentation. On the day of experimentation, the SFM was removed and 100 μL fresh SFM added per well, followed by β‐antagonist (in 10 μL, triplicate wells). The plates incubated for 5 h at 37°C. SFM and all drugs were then removed, 40 μL SFM added to each well, and the plates incubated for 1 h at 37°C. The plates were then transferred to a 65°C oven for 30 min (to destroy endogenous phosphatases), cooled, and 100 μL 5 mM p‐NPP in diethanolamine buffer added to each well. The plates were read on Dynatech MRX plate reader at 405 nM once the yellow color developed. Basal and response to a control maximum (10 μM isoprenaline) were measured in all plates.

### 2.7 Data Analysis

All data were plotted using Graphpad Prism 10.

### 2.8 Affinity of ^3^H‐CGP12177

The affinity (*K*_D_) of ^3^H‐CGP12177 was determined from saturation binding by plotting the specific binding (SB) of ^3^H‐CGP12177 using the non‐linear regression program Prism 10 using:

$$\mathrm{SB}=\frac{\left(A\times B_{\max}\right)}{\left(A+K_{D}\right)}$$

where *A* is the concentration of ^3^H‐CGP12177, *B*_max_ is the maximal specific binding, and *K*_D_ is the dissociation constant of ^3^H‐CGP12177.

### 2.9 Affinity of the β‐Antagonists

Other β‐antagonist affinities were determined from competition binding where a sigmoidal response curve was then fitted to the data and the IC_50_ (concentration required to inhibit 50% of the specific binding) determined using:

$$\%\text{uninhibited binding}=100-\frac{\left(100\times A\right)}{\left(A+{\mathrm{IC}}_{50}\right)}+\mathrm{NS}$$

where *A* is the concentration of the competing ligand, and NS is non‐specific binding.

From the IC_50_ and known concentration of ^3^H‐CGP12177, a *K*_D_ value (concentration at which half the receptors are bound) for competing ligands was calculated using the Cheng‐Prusoff equation:

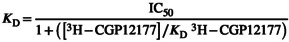




### 2.10 Duration of Binding of the Antagonists

For relative assessment of duration of binding, the rightward shift of the sigmoidal concentration response curve from the control to the wash plate (normalized to total and non‐specific binding for each plate) was noted [65]. Longer acting ligands that did not dissociate (during wash or subsequent 2 h ^3^H‐CGP12177 incubation) would result in similar ^3^H‐CGP12177 binding as control, whereas short duration ligands, removed during the wash and/or dissociating from the receptor during the 2 h ^3^H‐CGP12177 incubation, would result in more ^3^H‐CGP12177 binding and a larger rightward shift of the concentration response curve. Thus, the degree of rightward shift of the inhibition curve gives a relative measure of the duration of binding for that β‐antagonist. For most β‐antagonists, the washout curve was a parallel shift and the degree of this shift was noted.

For a few ligands, the washout curve was best described by a two‐component fit using the following equation:

$$\%\text{specific binding}=\frac{\left[A\right]\times N}{\left(\left[A\right]+{\mathrm{IC}}_{50}1\right)}+\frac{\left[A\right]\times \left(100-N\right)}{\left(\left[A\right]+{\mathrm{IC}}_{50}2\right)}$$

where [*A*] is the concentration of the competing ligand, IC_50_1 and IC_50_2 are the respective IC_50_ values for the two components, and *N* is the percentage of the response occurring through the first component (IC_50_1).

In these cases, the proportion (%) of the wash curve that was right‐shifted from the control curve and the degree of this shift was noted.

### 2.11 CRE‐SPAP Responses Gene Transcription Agonist Responses

Some ligands stimulated partial agonist responses best described by a one‐site sigmoidal concentration response curve:

$$\text{Response}=\frac{E_{\max}\times \left[A\right]}{{\mathrm{EC}}_{50}+\left[A\right]}$$

where *E*_max_ is the maximum response, [*A*] the agonist concentration, and EC_50_ the concentration of ligand that produces 50% of the maximal response.

Some ligands, however, stimulated a partial agonist response that was best described by a:

$$\%\text{maximal stimulation}=\frac{\left[A\right]\times N}{\left(\left[A\right]+\mathrm{EC}1_{50}\right)}+\frac{\left[A\right]\times \left(100-N\right)}{\left(\left[A\right]+\mathrm{EC}2_{50}\right)}$$

where *N* is the percentage of site 1, [*A*] is the concentration of agonist, and EC1_50_ and EC2_50_ are the respective EC_50_ values for the two agonist sites.

Basal and 10 μM isoprenaline concentration measured in every plate allowed agonist responses to be expressed as a percentage of the maximum response to isoprenaline.

## 3 Results

### 3.1 Affinity of ^3^H‐CGP12177 From Saturation Binding

The affinity of ^3^H‐CGP12177 was determined from saturation binding and found to be 0.22 ± 0.01 nM, 514 fmol/mg protein, *n* = 28 in the CHO‐β2 cells and 0.42 ± 0.02 nM, 1133 fmol/mg protein, *n* = 29 in the CHO‐β1 cells, very similar to that previously reported in these cell lines stably expressing the human receptors [64].

### 3.2 Affinity and Selectivity of β‐Antagonists for WT CHO‐β2 and CHO‐β1

**FIGURE 1:**
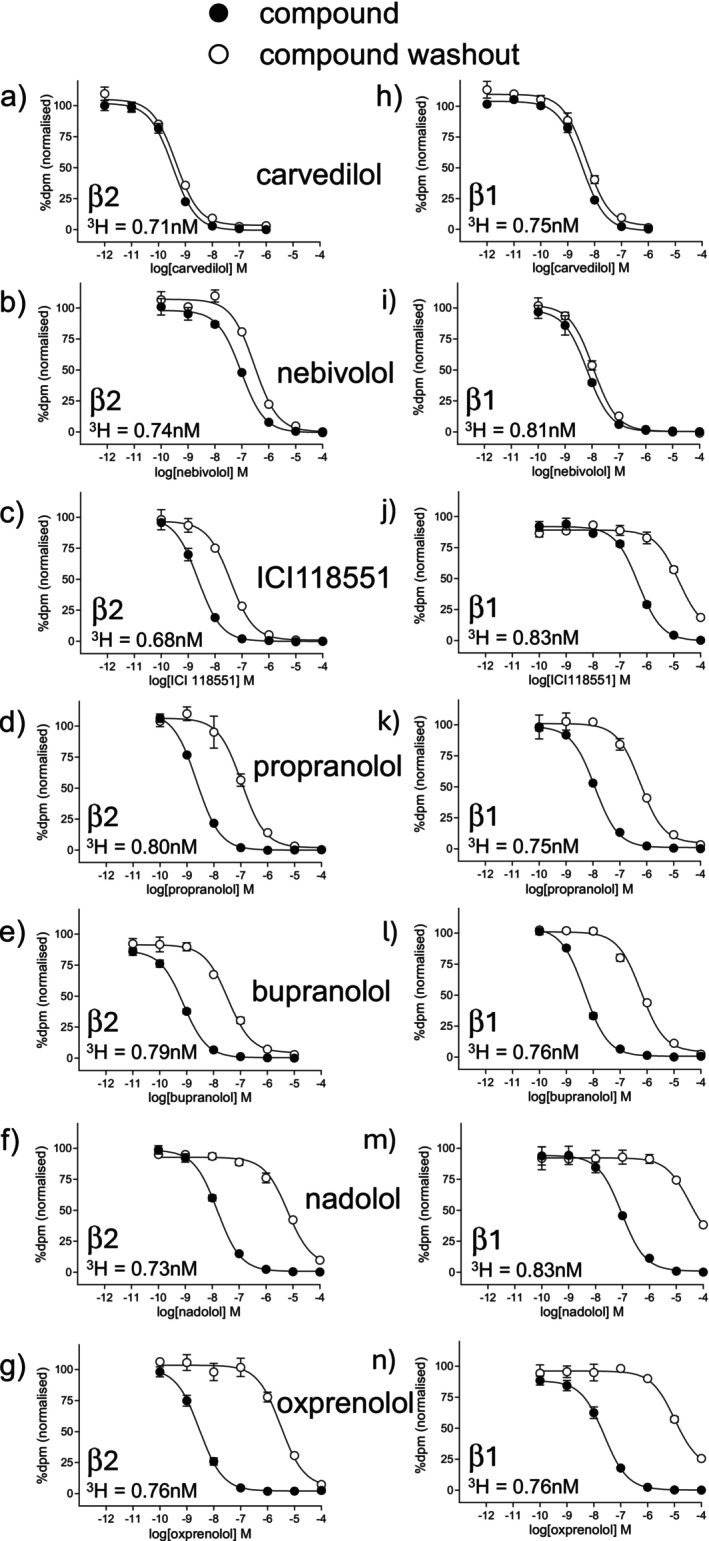
Inhibition of ^3^H‐CGP12177 whole cell specific binding in (a–g) CHO‐β2 cells and (h–n) CHO‐β1‐cells in response to (a) and (h) carvedilol, (b) and (i) nebivolol, (c) and (j) ICI118551, (d) and (k) propranolol, (e) and (l) bupranolol, (f) and (m) nadolol and (g) and (n) oxprenolol and following ligand washout. Non‐specific binding was determined by 10 μM propranolol and data points are mean ± SEM of triplicate determinations. Each graph is from a single experiment, representative of 10–25 separate experiments (see Table 2 for exact *n* numbers for each ligand). The concentration of ^3^H‐CGP12177 present in each experiment shown is given on each graph.

The affinity of 35 β‐antagonists was examined in the wildtype cell lines (Figure 1, Table 1). As expected, ICI118551, a known β2‐selective antagonist, had high affinity for the CHO‐β2 cell line (log *K*_D_ −9.37), 355‐fold higher than that in the CHO‐β1 cell line. Likewise CGP20712A, a known β1‐selective antagonist had 1259‐fold higher affinity in the CHO‐β1 cell line. This confirms the presence of the β2 and β1‐AR in the respective cell lines. Cyanopindolol had the highest affinity in both CHO‐β2 and CHO‐β1 cell lines, and 14 ligands were identified with sub‐nanomolar affinity at the β2‐AR. Their affinities and β2 vs. β1‐AR selectivities are given in Table 1, arranged in order of β2‐affinity, with the highest affinity compounds at the top of the table.

**TABLE 1:** Log *K*_D_ values for the affinity of β‐blockers measured from ^3^H‐CGP12177 whole cell binding in CHO‐β2 and CHO‐β1 cells. Values are mean ± SEM of *n* separate experiments. The β2 or β1 selectivity of compounds is also given (thus cyanopindolol is 3.6‐fold β2‐selective). Compounds are arranged in order of β2‐affinity.

	β2		β1		Selectivity	
	Log *K*_D_	*n*	Log *K*_D_	*n*	β2	β1
Cyanopindolol	−10.70 ± 0.05	13	−10.14 ± 0.06	13	3.6	
Carazolol	−10.38 ± 0.03	15	−9.68 ± 0.04	14	5.0	
Carpindolol	−10.25 ± 0.03	12	−9.00 ± 0.03	18	17.8	
Bucindolol	−10.14 ± 0.06	16	−9.44 ± 0.07	17	5.0	
Levobunolol	−10.09 ± 0.05	8	−8.37 ± 0.03	9	52.5	
Timolol	−10.02 ± 0.03	19	−8.73 ± 0.03	19	19.5	
Carvedilol	−9.94 ± 0.05	14	−9.19 ± 0.06	15	5.6	
Bupranolol	−9.92 ± 0.03	18	−8.78 ± 0.02	18	13.8	
Carteolol	−9.68 ± 0.08	10	−8.70 ± 0.06	14	9.5	
CGP12177	−9.56 ± 0.09	14	−9.32 ± 0.02	13	1.7	
ICI118551	−9.37 ± 0.02	24	−6.82 ± 0.02	29	354.8	
Propranolol	−9.27 ± 0.04	21	−8.32 ± 0.02	22	8.9	
Pindolol	−9.13 ± 0.04	12	−8.58 ± 0.08	13	3.5	
Alprenolol	−9.08 ± 0.04	11	−8.06 ± 0.04	10	10.5	
Oxprenolol	−8.99 ± 0.03	12	−7.97 ± 0.04	11	10.5	
BAAM	−8.74 ± 0.03	22	−7.99 ± 0.05	22	5.6	
Nadolol	−8.52 ± 0.04	13	−7.54 ± 0.03	13	9.5	
SR59230A	−8.23 ± 0.04	10	−7.53 ± 0.04	10	6.2	
Labetolol	−8.15 ± 0.05	12	−8.00 ± 0.05	12	1.4	
Nebivolol	−7.44 ± 0.05	12	−8.63 ± 0.06	13		15.5
Bevantolol	−7.30 ± 0.05	9	−7.91 ± 0.04	9		4.1
Pronethalol	−7.23 ± 0.04	10	−6.82 ± 0.02	10	2.6	
ZD7114	−7.18 ± 0.03	10	−7.58 ± 0.03	10		2.5
Betaxolol	−7.10 ± 0.03	11	−8.26 ± 0.06	11		14.5
ICI89406	−7.05 ± 0.03	9	−8.89 ± 0.04	9		69.2
Metoprolol	−6.72 ± 0.03	13	−7.65 ± 0.04	10		8.5
Sotalol	−6.66 ± 0.05	10	−5.89 ± 0.02	9	5.9	
Bisoprolol	−6.42 ± 0.05	17	−8.09 ± 0.03	17		46.8
Xamoterol	−5.89 ± 0.02	12	−7.28 ± 0.04	12		24.5
CGP20712A	−5.81 ± 0.03	18	−8.91 ± 0.05	21		1258.9
Acebutolol	−5.79 ± 0.03	11	−6.73 ± 0.03	11		8.7
NDD825	−5.59 ± 0.07	11	−8.24 ± 0.08	11		446.7
Atenolol	−5.57 ± 0.03	10	−6.99 ± 0.06	9		26.3
Landiolol	−5.03 ± 0.08	8	−6.95 ± 0.06	8		73.2
Practolol	−5.03 ± 0.06	7	−6.38 ± 0.04	8		22.4

### 3.3 Duration of Binding for WT CHO‐β2 and CHO‐β1

A relative measure of duration of action was then determined from the washout studies (Figure 1, Table 2). As can be seen in Figure 1, the washout process caused a log 2.85 (708‐fold) rightward shift of the specific binding curve for oxprenolol (Figure 1g,n), suggesting that oxprenolol was readily washed out and thus has a relatively short duration of receptor binding. With the same washout process, the curve for carvedilol was barely shifted at all, suggesting that carvedilol remained bound to the receptor (i.e., was longer acting, Figure 1a,h). Propranolol had a rightward shift of log 1.67 (47‐fold). Table 2 shows the rightward shift achieved for each compound, arranged in order of longevity for the β2‐AR with the longest acting compounds at the top of the table.

**TABLE 2:** Duration of binding as measured by a rightward shift of the ^3^H‐CGP12177 displacement curve following washout of β‐blocker. For ligands with a biphasic washout curve (Figure 2), the % of the response right‐shifted and shift of this component are given. Thus for carazolol at β1, 26.8% of the curve was shifted to the right by log 1.36 (23‐fold) fold (Figure 2e). For all other compounds, the shift was parallel (i.e., 100% was shifted, as in Figure 1). Values are mean ± SEM of *n* separate experiments. Compounds are arranged in order of duration of binding for the β2‐AR.

	β2			β1		
	Log shift	% Curve shifted	*n*	Log shift	% Curve shifted	*n*
Cyanopindolol	−0.07 ± 0.07		12	1.26 ± 0.07	9.0% ± 0.9%	13
Carazolol	0.08 ± 0.02		15	1.36 ± 0.04	26.8% ± 1.7%	14
Carvedilol	0.11 ± 0.05		13	0.33 ± 0.05		15
Nebivolol	0.47 ± 0.06		12	0.28 ± 0.08		13
SR59230A	0.50 ± 0.08		10	0.56 ± 0.05		10
BAAM	0.91 ± 0.04	30.2% ± 2.7%	19	1.17 ± 0.06	45.4% ± 1.6%	21
Bucindolol	1.19 ± 0.08	19.9% ± 1.4%	16	1.06 ± 0.04	39.1% ± 3.0%	17
ICI118551	1.21 ± 0.07		24	1.81 ± 0.05		25
Carpindolol	1.42 ± 0.08	11.7% ± 1.8%	11	2.16 ± 0.06	71.2% ± 2.1%	17
Propranolol	1.67 ± 0.07		21	1.68 ± 0.06		22
Bupranolol	1.86 ± 0.06		18	1.97 ± 0.04		15
Alprenolol	2.03 ± 0.06		11	2.31 ± 0.09		10
Bevantolol	2.05 ± 0.08		9	1.81 ± 0.10		9
Labetolol	2.21 ± 0.05		12	2.34 ± 0.08	61.5% ± 3.8%	10
Pronethalol	2.29 ± 0.09		10	2.78 ± 0.09		10
Carteolol	2.29 ± 0.09	48.6% ± 3.0%	10	2.67 ± 0.10	72.2% ± 2.1%	10
Levobunolol	2.32 ± 0.06	59.1% ± 4.2%	8	2.62 ± 0.09		9
ICI89406	2.62 ± 0.04		8	2.11 ± 0.06	69.4% ± 2.5%	9
Nadolol	2.68 ± 0.06		13	2.83 ± 0.0		12
Timolol	2.73 ± 0.06	49.9% ± 1.5%	18	2.89 ± 0.09	74.0% ± 2.0%	18
Bisoprolol	2.83 ± 0.08		8	2.64 ± 0.08		17
Pindolol	2.83 ± 0.08		12	2.89 ± 0.08		13
Oxprenolol	2.85 ± 0.06		12	2.91 ± 0.12		10
Betaxolol	2.88 ± 0.07		11	2.18 ± 0.06		11
Sotalol	2.96 ± 0.08		10	> 3		9
ZD7114	3.04 ± 0.05		10	2.74 ± 0.07		10
Metoprolol	3.04 ± 0.10		13	3.29 ± 0.07		9
CGP12177	3.13 ± 0.07	66.7% ± 1.8%	14	3.04 ± 0.06	63.1% ± 2.7%	13
Practolol	> 1		7	> 2		8
Landiolol	> 1		8	> 3		8
CGP20712A	> 1.5		18	1.68 ± 2.9	62.1% ± 2.9%	18
NDD825	> 1.5		11	1.77 ± 0.06	59.2% ± 3.4%	11
Acebutolol	> 2.5		11	> 3		10
Xamoterol	> 2.5		10	3.04 ± 0.07		11
Atenolol	> 2.5		10	3.41 ± 0.06		9

**FIGURE 2:**
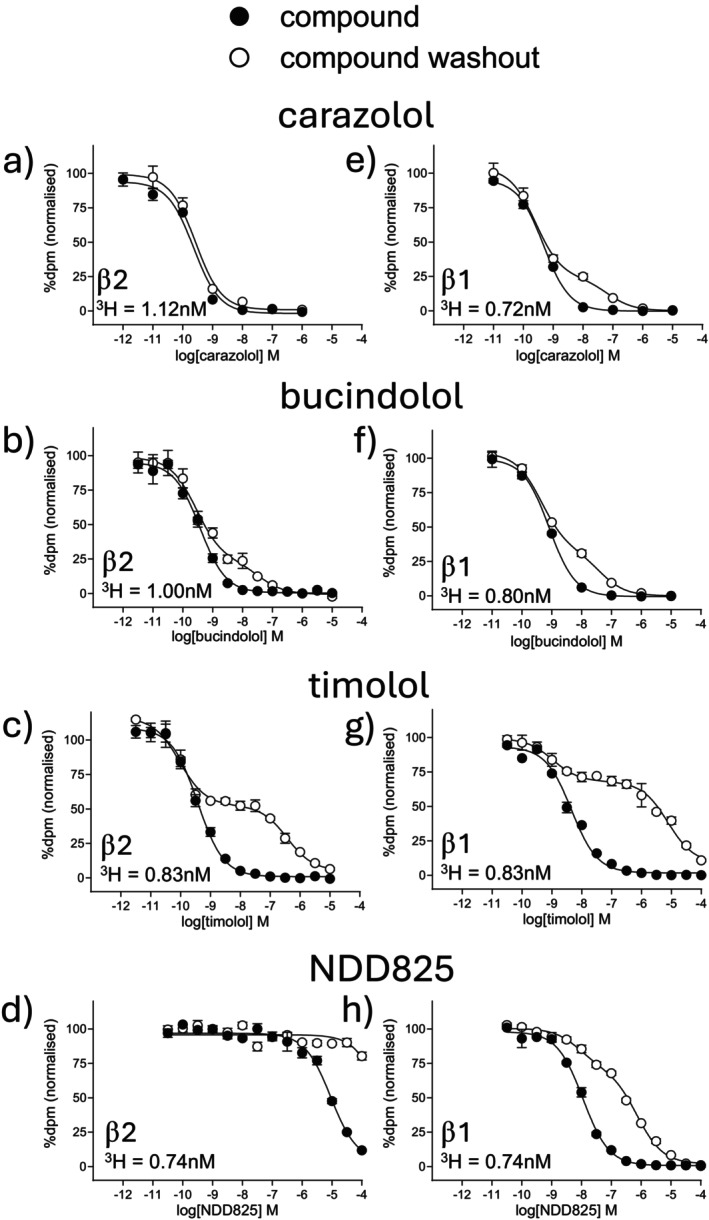
Inhibition of ^3^H‐CGP12177 whole cell specific binding in (a–d) CHO‐β2 cells and (e–h) CHO‐β1‐cells in response to (a) and (e) carazolol, (b) and (f) bucindolol, (c) and (g) timolol, and (d) and (h) NDD825 and following ligand washout. Non‐specific binding was determined by 10 μM propranolol and data points are mean ± SEM of triplicate determinations. Each graph is from a single experiment, representative of 11–18 separate experiments (see Table 2 for exact *n* numbers for each ligand). The concentration of ^3^H‐CGP12177 present in each experiment shown is given on each graph.

As can be seen from Figure 2 and Table 2, the inhibition of specific binding for a few compounds following washout was best described by a two‐component curve. In all cases, the curve followed that of control at lower concentrations but became more right‐shifted (washed out) at higher concentrations of β‐blocker. The proportion of that remaining bound and washed out varied between β‐blockers, with some only having a small proportion being washed out (e.g., carazolol Figure 2e) and others a large proportion washed out (e.g., timolol Figure 2g). As above, the rightward shift also varied between ligands, and the findings in the CHO‐β2 and CHO‐β1 cells were not identical. The reason for this apparent two‐component washout is unknown. Table 2 contains the rightward shift of the second (lower affinity) component and the proportion of the curve that was shifted (i.e., for carazolol in the CHO‐β1 cells, only 26.8% of the curve was shifted by 1.36 log units or 23‐fold, whereas for timolol, 74% of the curve was shifted by 2.89 log units, or 776‐fold).

### 3.4 Intrinsic Efficacy in WT CHO‐β2 and CHO‐β1—CRE‐SPAP Production

**FIGURE 3:**
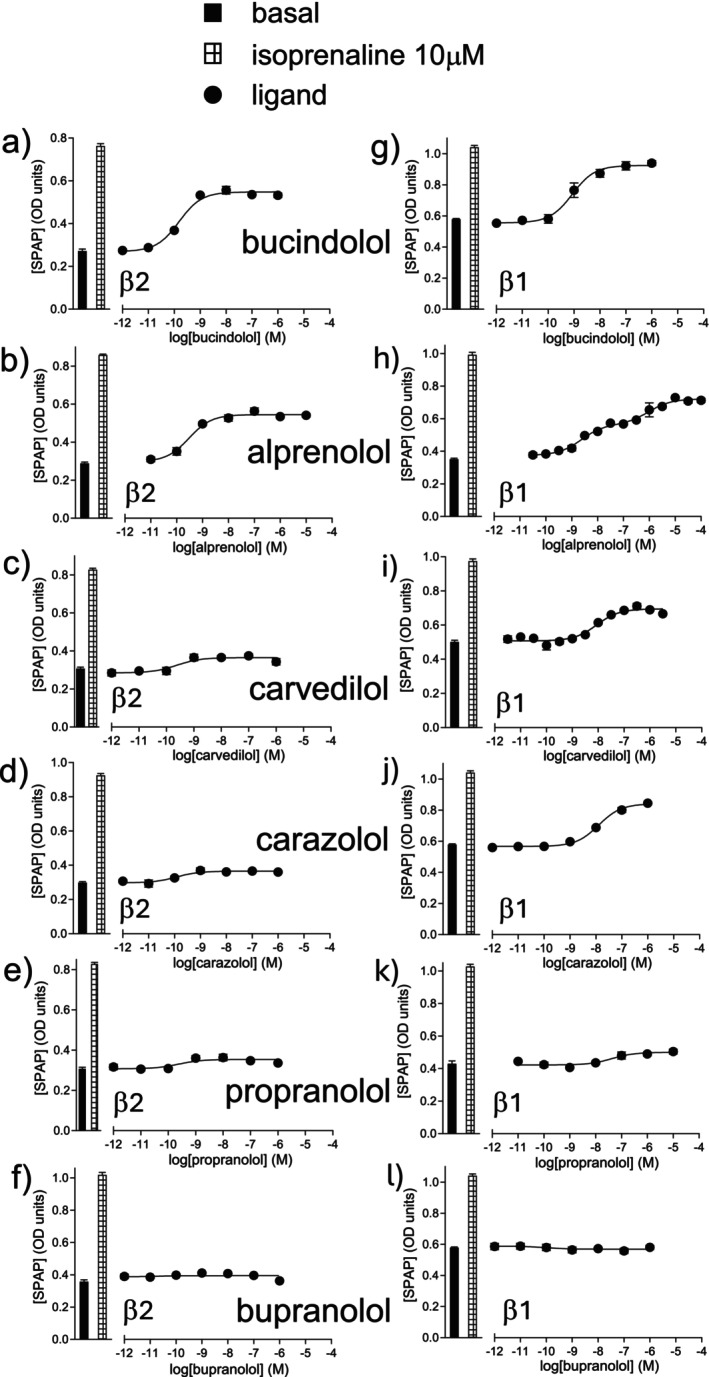
CRE‐SPAP production in (a–f) CHO‐β2 cells and (g–l) CHO‐β1 cells in response to (a) and (g) bucindolol, (b) and (h) alprenolol, (c) and (i) carvedilol, (d) and (j) carazolol, (e) and (k) propranolol and (f) and (l) bupranolol. Bars represent basal and CRE‐SPAP production in response to 10 μM isoprenaline. Data points are mean ± SEM of triplicate determinations and these single experiments are representative of 6–24 separate experiments (see Table 3 for exact *n* numbers for each ligand).

Several “β‐blockers” are known to have agonist actions at both the β2 and β1‐AR (e.g., carvedilol stimulates a small increase in both cAMP and CRE‐gene transcription in these cell lines [60, 61, 67]) so the stimulatory agonist effects were studied in both cell lines. The CRE‐SPAP response was measured as this a mixed read out from both cAMP‐mediated CRE‐gene transcription and ERK1/2 MAPKinase‐mediated gene transcription, and given the amplification, allows small responses to be more readily measured [60] and thus compounds ranked in order of efficacy. Many “β‐blockers” stimulated agonist responses (Figure 3, Table 3). Table 3 shows the log EC_50_ and % isoprenaline of the responses obtained, arranged in order of β2‐efficacy. As can be seen, the order of efficacy is not the same for β2‐AR and β1‐AR.

**TABLE 3:** Log EC_50_ and % response compared to that of 10 μM isoprenaline from CRE‐SPAP production in CHO‐β2 and CHO‐β1 cells. For certain compounds a biphasic response was seen (e.g., Figure 3h) so log EC_50_ at component 1 (EC_50_1) and component 2 (EC_50_2) are given with the % of the response occurring via component 1, and % isoprenaline for the whole response. Values are mean ± SEM of *n* separate experiments. Compounds are arranged in order of β2‐efficacy (size of response).

	β2			β1				
	Log EC_50_	% Isop	*n*	Log EC_50_1	Log EC_50_2	% Site 1	% Isop	*n*
Labetolol	−8.64 ± 0.07	68.0 ± 4.1	12	−8.21 ± 0.06	−6.00 ± 0.12	53.2 ± 2.4	59.9 ± 2.9	7
Bucindolol	−9.75 ± 0.04	66.6 ± 2.8	11	−9.08 ± 0.07			78.5 ± 5.9	11
Pindolol	−9.70 ± 0.04	50.8 ± 2.8	11	−8.54 ± 0.10	−6.16 ± 0.10	51.5 ± 2.7	85.5 ± 3.6	16
Acebutolol	−6.52 ± 0.04	48.5 ± 3.4	11	−7.13 ± 0.11			56.8 ± 2.0	10
Cyanopindolol	−9.94 ± 0.06	44.6 ± 1.6	13	−8.80 ± 0.11			73.2 ± 4.4	12
Carteolol	−9.53 ± 0.10	44.2 ± 4.9	9	−8.75 ± 0.09	−6.22 ± 0.10	51.8 ± 3.5	75.2 ± 6.0	19
CGP12177	−9.57 ± 0.05	43.5 ± 1.8	21	−8.49 ± 0.05			84.3 ± 2.5	20
Alprenolol	−9.59 ± 0.04	41.3 ± 2.2	14	−8.54 ± 0.09	−6.22 ± 0.16	51.8 ± 2.8	65.3 ± 4.2	13
Practolol	10 μM	40.2 ± 5.6	6	−7.10 ± 0.06			58.7 ± 4.7	14
Oxprenolol	−9.61 ± 0.11	38.5 ± 3.5	11	−8.39 ± 0.08	−5.87 ± 0.25	76.7 ± 4.1	75.8 ± 4.5	9
Pronethalol	−8.01 ± 0.07	38.4 ± 2.4	14	−7.52 ± 0.08			77.6 ± 3.0	6
Xamoterol	−6.54 ± 0.07	37.9 ± 3.8	15	−8.60 ± 0.07			93.8 ± 6.8	6
ICI89406	−7.30 ± 0.06	31.3 ± 1.9	17	−9.33 ± 0.04			74.3 ± 4.3	22
BAAM	−8.81 ± 0.11	27.2 ± 1.9	9	−7.82 ± 0.07			67.7 ± 1.8	9
Carpindolol	−9.64 ± 0.08	23.4 ± 3.2	6	−9.06 ± 0.05	−6.85 ± 0.06	51.2 ± 2.9	82.9 ± 3.9	10
Carvedilol	−9.35 ± 0.09	18.5 ± 1.5	20	−7.93 ± 0.10			33.3 ± 3.5	14
SR59230A	−8.26 ± 0.07	17.4 ± 1.4	10	−7.66 ± 0.05			82.7 ± 5.4	7
ZD7114	−7.59 ± 0.08	14.8 ± 1.5	16	−8.62 ± 0.08			92.2 ± 5.4	6
CGP20712A	−5.83 ± 0.16	11.3 ± 2.2	18	No response				13
Carazolol	−10.14 ± 0.15	9.7 ± 1.4	9	−7.89 ± 0.06			58.7 ± 4.3	16
Propranolol	−9.66 ± 0.10	9.2 ± 0.8	19	−6.88 ± 0.14			15.0 ± 3.5	24
Bevantolol	No response		9	−7.78 ± 0.09			46.2 ± 6.9	9
Atenolol	No response		5	No response				5
Betaxolol	No response		5	No response				8
Bisoprolol	No response		5	No response				5
Bupranolol	No response		7	No response				6
ICI118551	No response		5	No response				6
Landiolol	No response		7	No response				8
Levobunolol	No response		6	No response				6
Metoprolol	No response		5	No response				5
Nadolol	No response		7	No response				10
NDD825	No response		6	No response				6
Nebivolol	No response		8	No response				8
Sotalol	No response		5	No response				5
Timolol	No response		6	No response				7

A few ligands (e.g., alprenolol, Figure 3h) exhibited biphasic concentration responses at the β1‐AR. This was expected (see Ref. [61, 67, 68, 69, 70]). The β1‐AR exists in at least two active conformations: a catecholamine conformation where catecholamines, most other agonists and β‐blockers interact, and a separate secondary conformation where certain compounds stimulate agonist responses but at higher concentrations than those required to occupy the catecholamine conformation (e.g., Ref. [61, 70, 71, 72, 73, 74]). For a conventional partial agonist, the concentration required to stimulate half of its maximum response (EC_50_ value) should occur when half of the receptors are occupied (*K*_D_ value). As ligand efficacy increases, the EC_50_ value will become progressively lower (left‐shifted) than the *K*_D_ as fewer receptors need to be occupied to stimulate a more maximal response. Due to the low concentration of ^3^H‐CGP12177 used, the binding assay used above would only detect catecholamine conformation affinity. In keeping with previous studies [61, 69, 73], several ligands (e.g., acebutolol, ICI89406, practolol, pronethalol, xamoterol and ZD7114) behaved as typical β1‐partial agonists (EC_50_ lower/more potent than *K*_D_), suggesting catecholamine conformation activation. Again as previously, some compounds had high affinity as measured from the binding studies (with no stimulatory effects at this concentration), but a stimulatory response was seen at significantly higher compound concentrations (EC_50_ greater than *K*_D_). For example, carazolol (Figure 2e) has a β1‐affinity of log *K*_D_ −9.68 (0.2 nM), and at this concentration, no β1‐stimulatory response is seen (Figure 3j). However, at higher concentrations, an agonist response is observed (log EC_50_ −7.89, 12.9 nM; Figure 3j). This is not reconcilable with interaction at a single site or conformation. These agonist responses (e.g., CGP12177, carazolol, carvedilol and propranolol) were occurring via a secondary conformation [61, 70]. Finally, as previously observed, several ligands stimulate both conformations, resulting in biphasic responses (e.g., alprenolol, carpindolol, carteolol, labetolol, oxprenolol and pindolol [61, 67, 68, 69, 70]).

### 3.5 Investigation of Affinity and Efficacy at the β2 and β1‐Polymorphic Variants

As there are several naturally occurring polymorphisms (point mutations) in both the β2 and β1‐AR, it was important to determine whether the findings in wildtype receptors are applicable to all β2 and β1‐AR. A selection of compounds with at least 2 out of 3 favorable characteristics (affinity, duration, low efficacy) were therefore studied in cells stably expressing each of the polymorphic variants. The affinity of ^3^H‐CGP12177 in the polymorphic cell lines has previously been determined as very similar to that in the wildtype β‐ARs [62, 63] and, together with receptor expression levels, is summarized in Table S4.

As previously shown, the threonine at β2‐164 is important for the affinity of ICI118551 (for both the isoleucine substitution in the natural polymorphism [62] or a valine substitution as in the β1‐AR [75]). ICI118551, once again, had 7.6‐fold lower affinity for β2‐ile164 than the wildtype β2‐AR or the other polymorphisms (Figure S1, Table S2). Interesting, bupranolol also has 8.3 fold lower affinity in this β2‐polymorphism (Figure S1, Table S2), demonstrating that it is important to check the effect of polymorphisms compared with wild type receptor when considering clinical use. As expected, the overall level of response was dependent on the receptor expression level, with lines with low receptor expression having few measurable responses, and CHO‐β1‐arg389 having several measurable responses; however, the rank order of compounds was the same as wildtype, suggesting no significant changes in efficacy (Figure S2, Table S3). Thus, with the exception of ICI118551 and bupranolol at β2‐ile164, the pharmacological profile of the other β‐blockers was similar to the β2 or β1‐wildtype.

## 4 Discussion

There is increasing evidence that β‐blockers appear to reduce cancer growth and metastasis, with propranolol the most investigated compound. However, β‐blockers have different molecular pharmacological properties that alter cardiovascular outcomes. For an “anti‐cancer” β‐blocker, theoretically, high β2‐affinity, long duration receptor binding and no agonism would be “ideal” to minimize endogenous catecholamine activation. Given the existence of many off‐patent β‐blockers, financial incentives to conduct comparative clinical trials are low. This study examined the affinity, duration of binding and efficacy of 35 β‐blockers to determine which existing β‐blocker has the most theoretically “ideal” anti‐cancer molecular pharmacological characteristics, and therefore a potential best candidate for future clinical trials.

High β2‐AR affinity is the first “ideal” characteristic. Fourteen compounds had sub‐nanomolar human β2‐AR affinity, of which 11 had higher β2‐affinity than propranolol, namely cyanopindolol, carazolol, carpindolol, bucindolol, levobunolol, timolol, carvedilol, bupranolol, carteolol, CGP12177, and ICI118551. With the exception of ICI118551, the other 13 compounds also had higher β1‐affinity than propranolol.

The second “ideal” characteristic is long duration receptor binding to prevent any endogenous catecholamine receptor occupancy driving cancer growth and metastasis. A longer‐acting drug at the receptor level may also buffer risks of undertreatment should plasma levels fall following a missed or delayed dose. In washout studies, nine β‐blockers had longer binding durations than propranolol. Of these nine, six also had higher affinity than propranolol. Thus, cyanopindolol, carazolol, carpindolol, bucindolol, carvedilol, and ICI118551 may have more optimal β‐AR affinity and duration than propranolol for an anti‐cancer agent. Of note, several compounds' washout curve was best described by a two‐component fit. The findings were not identical for β2‐AR and β1‐ARs. No ^3^H‐CGP12177 binding or functional responses occur in the absence of transfected receptors (e.g., Ref. [60, 61, 69, 70]), so the explanation for these biphasic washout responses remains unknown.

**FIGURE 4:**
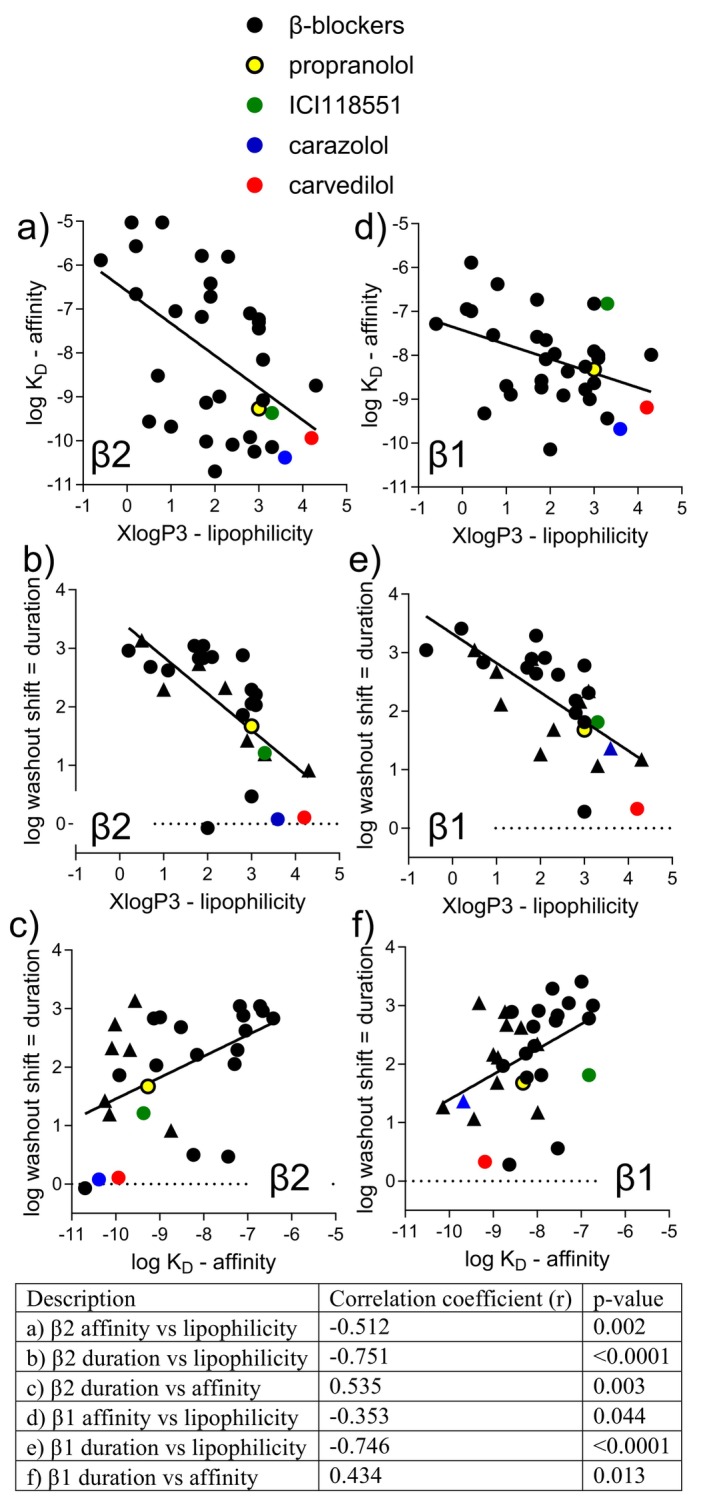
Correlation plots for (a–c) β2 and (d–f) β1 for (a) and (d) lipophilicity and affinity, (b) and (e) lipophilicity and duration of binding and (c) and (f) affinity and duration of binding. Lipophilicity is XlogP taken from PubChem https://pubchem.ncbi.nlm.nih.gov/docs/ (see Table S1), affinity is *K*_D_ value (from Table 1) and duration of binding is the rightward shift in washout assays, from Table 2. Propranolol is highlighted in yellow, ICI118551 in green, carazolol in blue and carvedilol in red. In (b), (c), (e) and (f) if the compound had a biphasic washout (Table 2), it is shown as a triangle with the shift of the second component plotted. Using a Spearman correlation (with no assumptions of normal distribution of data) the correlation coefficient *r* and *p*‐value are provided.

Binding longevity can be achieved in different ways. Compounds could be anchored to the receptor via an exosite and thus unable to diffuse away, even if the head‐group dissociated from the binding site, making head‐group rebinding highly likely (exosite theory e.g., Ref. [76]). This could however allow catecholamines to compete with the head‐group and allow unwanted stimulation. Lipophilic compounds could accumulate in cell membranes forming a reservoir that slowly leaches out to bind to receptors giving an overall long duration of action (microkinetic theory e.g., Ref. [77]). Alternatively, compounds could have a long receptor residence time within the binding pocket due to ligand‐receptor interactions within the binding pocket or entry/exit into the pocket (“sticky ligand”). A recent study examining the binding duration of β2‐agonists for asthma and COPD found that salmeterol and vilanterol utilize a specific β2‐AR exosite resulting in very high affinity. Mutation of this exosite drastically reduced affinity but the long duration remained. Binding duration was closely correlated with lipophilicity, making the microkinetic theory the most likely for β2‐agonist duration [62]. Although statistically significant, for β‐blockers, the correlation between affinity and lipophilicity is poor, as is affinity and duration of binding (Figure 4). There is a better correlation between lipophilicity and duration of binding, although the scatter suggests lipophilicity is not the sole explanation for the duration of binding of β‐blockers.

As β‐agonism promotes cancer growth and metastasis, an “ideal” anti‐cancer β‐blocker should have no agonism. Whilst a well‐recognized and frequent criticism of high receptor expressing transfected cells is that small and clinically insignificant responses can be detected, examining such responses allows compounds to be ranked in order of agonism. The question is at what level does partial agonism detected in cell systems stop being clinically relevant. Considering the cardiovascular field, compounds with little or no partial agonism reduced mortality (carvedilol, propranolol, nebivolol, metoprolol, bisoprolol), whilst compounds with significant partial agonism were unhelpful (oxprenolol, pindolol, bucindolol), and xamoterol with even higher partial agonism was detrimental. A linear correlation was found between partial agonism measured in these CHO‐β1 cells and resting heart rate in conscious rats. Labetolol and ligands with partial agonism below labetolol in the cell line (e.g., ICI118551, bisoprolol, nebivolol and carvedilol) caused a reduction in rat resting heart rate whereas ligands with higher partial agonism (e.g., xamoterol, bucindolol, pronethalol and acebutolol) caused an increase in resting heart rate [67]. Notably, carvedilol (low partial agonism) reduced heart rate in rats and clinically was as successful in reducing heart failure mortality as the neutral antagonists bisoprolol and metoprolol [54, 55, 56], suggesting that carvedilol's partial agonism is below that of clinical relevance. Thus, for the cardiovascular system, clinical trials and animal studies suggest that labetolol might be the ceiling for physiologically detectable/relevant partial agonism, and labetolol and compounds with less partial agonism than labetolol appearing clinically as neutral antagonists.

Few β‐blockers have been studied in cancer to determine the “safe level” of partial agonism. Labetolol appeared as effective as propranolol in reducing tumor burden in rats, albeit under stress conditions that may raise baseline tumor growth, suggesting this partial agonism is, at worst, not detrimental [28]. Carvedilol did not stimulate agonist responses in breast cancer cells, inhibited stress‐induced tumor growth and metastasis, and appeared beneficial in reducing mortality in women with breast cancer [23]. Carvedilol use was associated with a 26% reduction in cancer incidence in a large population study [43]. Propranolol has frequently been reported helpful. Thus, the partial agonist activity of propranolol and carvedilol (and maybe labetolol) appears below that clinically meaningful for cancer. Thus, keeping partial agonism at or below labetalol would seem important for both long‐term cancer and cardiovascular outcomes.

Here, labetolol had substantial β2‐partial agonism but was by no means the most efficacious β1‐ligand. Of the 6 ligands identified from binding studies, cyanopindolol, carpindolol, and bucindolol had higher β1‐AR partial agonism than labetolol, suggesting more potential long‐term cardiovascular risk (regardless of their effects in cancer). Bucindolol and cyanopindolol also had substantial β2‐partial agonism. This leaves carazolol, carvedilol, and ICI118551, with high affinity, long duration, and low partial agonism, as potential better alternatives to propranolol for cancer.

Whilst propranolol, carazolol, and carvedilol all stimulated some β1‐partial agonism, for all 3 compounds this is occurring via the secondary conformation. Although it can clearly be demonstrated in blood vessels and whole animals (see Ref. [70] and references therein), the physiological (or pathological) role of secondary conformation responses remains unknown for both cardiovascular and cancer outcomes. Studies with the polymorphic variants of the β2 and β1‐AR revealed that although ICI118551 and bupranolol had slightly lower affinity for the rarest polymorphism (β2‐ile164 [62]), the other compounds, including propranolol, carazolol, ICI118551, and carvedilol, all have the same pharmacological profile as for wildtype β2 and β1‐AR meaning there would be no need to genotype people before offering treatment.

Thus three compounds were identified with potentially more optimal “anti‐cancer” characteristics than propranolol: carazolol, ICI118551 and carvedilol. Carazolol's high affinity and long duration make it attractive. It has similar β2‐partial agonism to propranolol, although greater β1‐partial agonism (similar to labetolol), however it was not clinically developed. ICI118551 has high β2‐AR affinity and longer duration than propranolol without any partial agonism. It is very β2‐selective, potentially less effective in certain β1‐AR expressing cancers, but was also not developed for clinical use.

Carvedilol, however, has very favorable molecular pharmacological characteristics: higher β2 and β1‐AR affinity and longer duration of binding than propranolol, whilst maintaining low partial agonism. Carvedilol has a long history of clinical use and remains one of the most frequently prescribed β‐blockers for cardiovascular conditions. It has a long‐term safety record and is already in widescale manufacture as an off‐patent oral medication (with once and twice daily regimes). It appeared helpful in breast cancer [23] and reduced the incidence of new cancers [43]. Beyond molecular pharmacological characteristics, carvedilol has a longer plasma half‐life than propranolol (7–10 h vs. 3–5 h [12]) which may have additional benefit if missed or delayed doses, and one of the highest affinities for β‐blockers at human β3‐ARs [64]. This study suggests that carvedilol should be considered as a potentially “better” β‐blocker for future clinical cancer studies than propranolol, albeit still contraindicated in those with asthma.

Retrospective epidemiological cancer studies often segregate β‐blockers into “selective” and “non‐selective” based on β1‐selectivity (e.g., Ref. [14] although not all studies list individual drugs). However, except for ICI118551, the selectivity of most β‐blockers examined in cancer studies is rather marginal (as in Ref. [64]). Despite this, many studies suggest that “non‐selective” β‐blockers appear more effective than “β1‐selectve” β‐blockers in cancer highlighting the β2‐requirement. However, “selectivity” groups often contain both neutral antagonists and those with significant partial agonism, for example, pindolol, alprenolol, oxprenolol, acebutolol, etc. To better understand which β‐blockers are most helpful in clinical cancer studies, it may be helpful to stratify drugs in epidemiological studies for partial agonism as well as selectivity.

In conclusion, this study suggests that carvedilol may, in theory, have more optimal molecular pharmacological characteristics for an “anti‐cancer” β‐blocker than propranolol. Carvedilol has a higher affinity and substantially longer duration of β2 (and β1) receptor binding than propranolol whilst maintaining low partial agonism and is readily available as an off‐patent tablet. Finally, whilst carvedilol and propranolol could be compared in more laboratory studies, given the already proven long‐term safety of carvedilol and propranolol and their potential benefit in cancer, it is time for prospective real‐world studies of these β‐blockers in people newly diagnosed with cancer [10, 47].

## Author Contributions

J.G.B. conceived the idea, performed all the experiments and data analysis and wrote the manuscript.

## Funding

This work was supported by the University of Nottingham and a Wellcome Trust Seeding Drug Discovery award (grant number 086039/Z/08/Z).

## Conflicts of Interest

The author declares no conflicts of interest.

## Supporting information


**Appendix S1:** prp270214‐sup‐0001‐AppendixS1.docx.

## Data Availability

Data available on request from the author.
