# Supplementary material for: Comparing the Molecular Pharmacological Properties of Existing β‐Blockers to Determine the Theoretically Most “Ideal” Anti‐Cancer β‐Blocker
Source: Pharmacol Res Perspect. 2026 Jan 3;14(1):e70214. doi: 10.1002/prp2.70214 (PMC12764437; doi:10.1002/prp2.70214)
Supplement: Supplementary file 1 — Appendix S1: prp270214‐sup‐0001‐AppendixS1.docx. [file PRP2-14-e70214-s001.docx]

**Comparing the molecular pharmacological properties of existing β-blockers to determine the most “ideal” anti-cancer β-blocker.**

Supplementary information Table S1 – source of ligands and lipohilicity

|  | source | product number | lipophilicity | |
| --- | --- | --- | --- | --- |
| acebutolol | Sigma | A3669 | XLogP3 | 1.7 |
| alprenolol | Sigma | A8676 | XLogP3 | 3.1 |
| atenolol | Sigma | A7655 | XLogP3 | 0.2 |
| BAAM (bromoacetyl  alprenolol menthane) | Sigma | B015 | XLogP3-AA | 4.3 |
| betaxolol | Tocris | 0906 | XLogP3 | 2.8 |
| bevantolol | Selleckchem | S5042 | XLogP3 | 3.0 |
| bisoprolol | Sigma | B2185 | XLogP3 | 1.9 |
| bucindolol | Tocris | 2658 | XLogP3-AA | 3.3 |
| bupranolol | Gift Sian Harding |  | XLogP3 | 2.8 |
| carazolol | Sigma | 53787 | XlogP3 | 3.6 |
| carpindolol (SDZ21009) | Tocris | 1516 | XLogP3-AA | 2.9 |
| carteolol | Selleckchem | S4278 | XLogP3-AA | 1.0 |
| carvedilol | Tocris | 2685 | XLogP3 | 4.2 |
| CGP12177 | Sigma | C125 | XLogP3-AA | 0.5 |
| CGP20712A | Tocris | 1024 | XLogP3-AA | 2.3 |
| cyanopindolol | Tocris | 0993 | XLogP3 | 2 |
| ICI118551 | Sigma | I127 | XLogP3-AA | 3.3 |
| ICI89406 | Tocris | 0832 | XLogP3 | 1.1 |
| labetolol | Sigma | L1011 | XLogP3 | 3.1 |
| landiolol | Sigma | SML1785 | XLogP3-AA | 0.8 |
| levobunolol | MedChemExpress | P-588278446 | XLogP3 | 2.4 |
| metoprolol | Sigma | M5391 | XLogP3 | 1.9 |
| nadolol | Sigma | N1892 | XLogP3 | 0.7 |
| NDD825 | Baker et al., 2017 |  |  |  |
| nebivolol | Sequoia | SRP035255n | XLogP3-AA | 3.0 |
| oxprenolol | Tocris | 3288 | XLogP3 | 2.1 |
| pindolol | Sigma | P0778 | XLogP3 | 1.8 |
| practolol | Tocris | 0831 | XLogP3 | 0.8 |
| pronethalol | Tocis | 0829 | XLogP3 | 3.0 |
| propranolol | Sigma | P0884 | XLogP3 | 3.0 |
| sotalol | Tocris | 0952 | XLogP3 | 0.2 |
| SR59230A | Tocris | 1511 |  |  |
| timolol | Sigma | T6394 | XLogP3 | 1.8 |
| xamoterol | Tocris | 0950 | XLogP3-AA | -0.6 |
| ZD7114 | Tocris | 0930 | XLogP3 | 1.7 |

Lipophilicity XlogP taken from PubChem <https://pubchem.ncbi.nlm.nih.gov/docs/> on 29^th^ September 2025. There is no XLogP3 for SR59230A given in Pubchem.

NDD825 is from Baker et al., (2017) FASEB J. 31: 3150-3166.

Bupranolol was a gift from Prof Sian Harding, Imperial College London. All data in the Tables is with this compound. To check, bupranolol was also bought from ADOOQ bioscience (product code A13400 14556-46-8). It had a β2-affinity log K_D_ -9.67 ± 0.07, log shift of 2.02 ± 0.12 in the washout (n=6) and at β1 log K_D_ -8.50 ± 0.05 washout log shift of 2.21 ± 0.08 (n=6). This confirms that despite high β2-affinity, bupranolol is readily washed out i.e. is short-acting.

Supplementary information Figure S1

Inhibition of ^3^H-CGP12177 whole cell specific binding in the naturally occurring polymorphic variants of the human β2 and β1-AR, in response to carazolol, carvedilol, propranolol and bupranolol. Control curves for CHO-β2-gly16, CHO-β2-gln27, CHO-β2-met34, CHO-β2-ile164, CHO-β1-gly49 and CHO-β1-arg389 is shown and those measured in parallel following washout of the β-blocker. Non-specific binding was determined by 10µM propranolol and data points are mean ± s.e.mean of triplicate determinations. Each graph is from a single experiment and is representative of 6-13 separate experiments as detailed in Tables S2a and S2b. The concentration of ^3^H-CGP12177 used in that experiment is given on each graph.


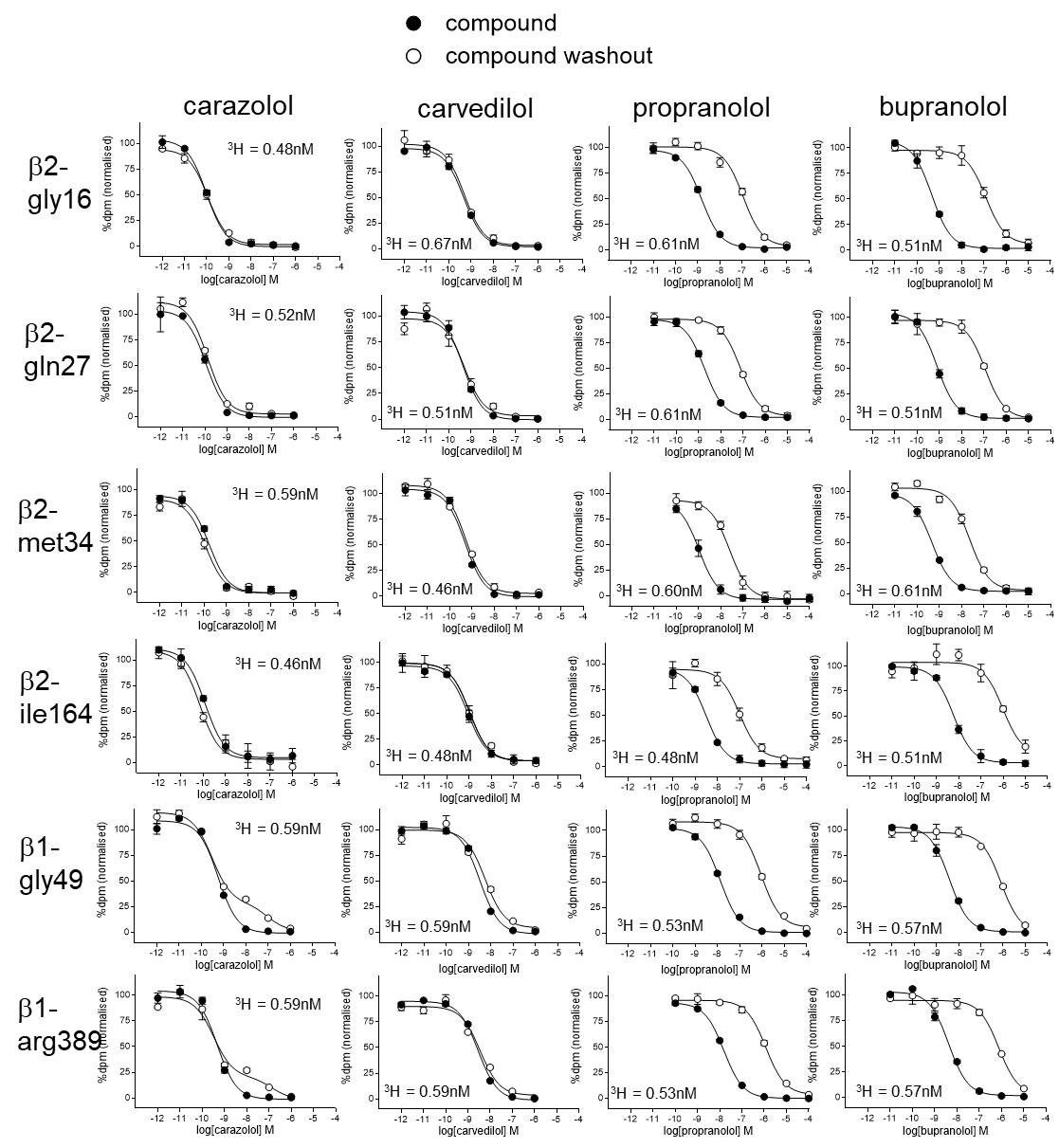


Supplementary information Figure S2

CRE-SPAP production in the naturally occurring polymorphic variants of the human β2 and β1-AR (CHO-β2-gly16, CHO-β2-gln27, CHO-β2-met34, CHO-β2-ile164, CHO-β1-gly49 and CHO-β1-arg389) in response to bucindolol, carvedilol and propranolol. Bars represent basal CRE-SPAP production and that in response to 10μM isoprenaline. Data points are mean ± sem of triplicate determinations and each graph is from a single experiment that is representative of 8-12 separate experiments, as detailed in Tables S3a and S3b.


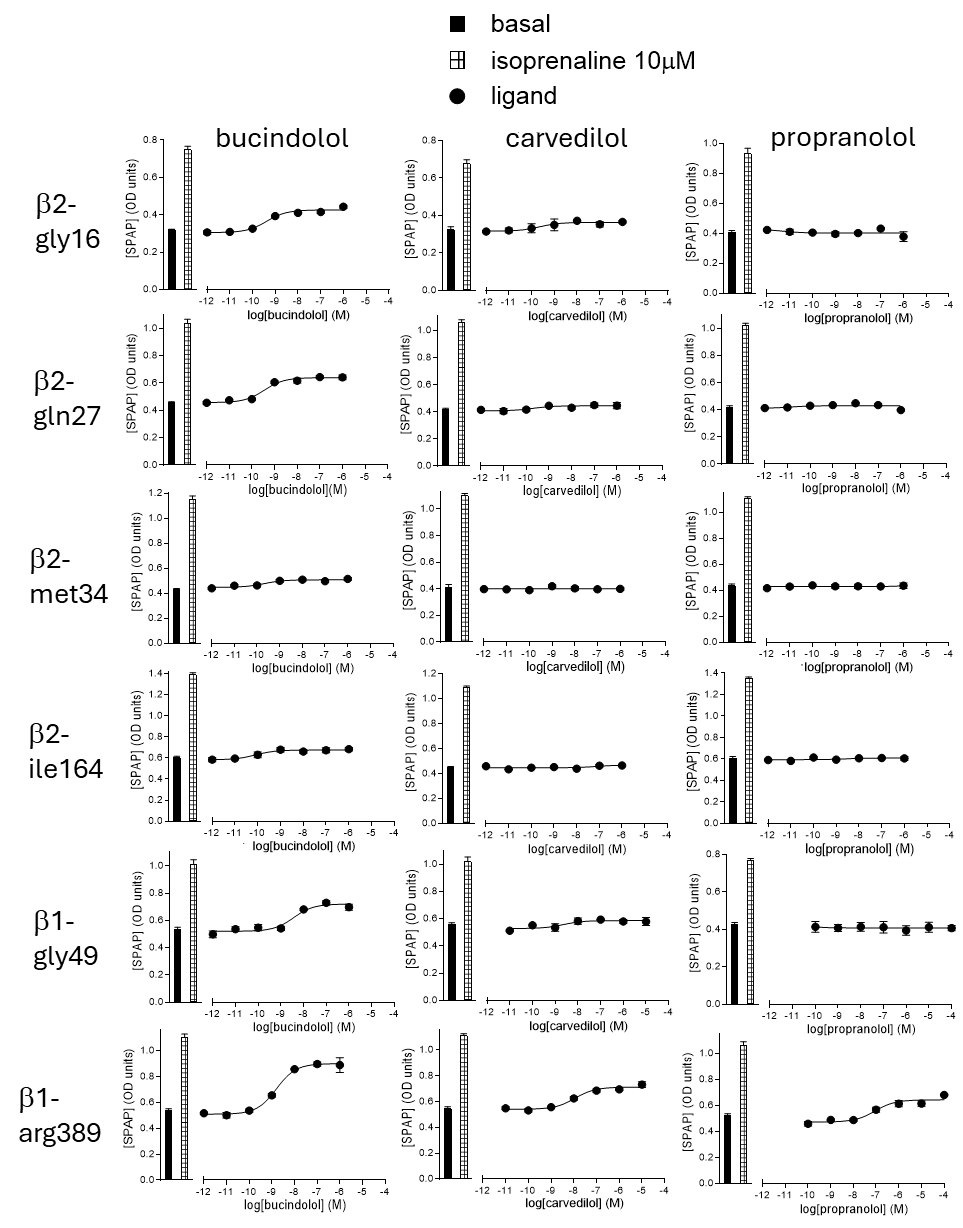


Supplementary information Table S2a and S2b

Log K_D_ values for the affinity of β-blockers measured from ^3^H-CGP12177 whole cell binding in cell lines stably expressing the naturally occurring polymorphic β-AR variants (CHO-β2-gly16, CHO-β2-gln27, CHO-β2-met34, CHO-β2-ile164, CHO-β1-gly49 and CHO-β1-arg389). The rightward shift following washout (log shift) is also given. Where compounds had a biphasic washout curve, the % of the curve shifted and log shift of that part of the curve is given (as in Table 2 of the main manuscript). Values are mean ± sem of n separate experiments. β2-AR and β1-AR wildtype data is taken from Tables 1 and 2. Compounds are arranged in order of β2-affinity (as for Table 1 of the main manuscript).

Table S2a – β2-AR polymorphic variants

|  | β2 wildtype | | CHO-β2-gly16 | | | CHO-β2-gln27 | | | CHO-β2-met34 | | | CHO-β2-ile164 | | |
| --- | --- | --- | --- | --- | --- | --- | --- | --- | --- | --- | --- | --- | --- | --- |
|  | Log K_D_ | Log shift | Log K_D_ | Log shift | n | Log K_D_ | Log shift | n | Log K_D_ | Log shift | n | Log K_D_ | Log shift | n |
| cyanopindolol | -10.70 | -0.07 | -10.62 ± 0.07 | 1.22 ± 0.18  13.3 ± 3.8% | 7 | -10.70 ± 0.03 | 1.08 ± 0.19  11.3 ± 2.4% | 7 | -10.92 ± 0.03 | -0.23 ± 0.08 | 6 | -10.80 ± 0.06 | -0.23 ± 0.06 | 7 |
| carazolol | -10.38 | 0.08 | -10.56 ± 0.04 | -0.11 ± 0.09 | 6 | -10.62 ± 0.08 | 0.05 ± 0.06 | 6 | -10.66 ± 0.07 | -0.12 ± 0.07 | 9 | -10.86 ± 0.07* | -0.07 ± 0.08 | 7 |
| carpindolol | -10.25 | 1.42  11.7 | -10.39 ± 0.05 | 1.09 ± 0.13  11.1 ± 0.30% | 8 | -10.40 ± 0.04 | 0.71 ± 0.17  6.1 ± 1.8 | 8 | -10.45 ± 0.04 | 1.08 ± 0.23  13.2 ± 2.7 | 7 | -10.39 ± 0.04 | 0.68 ± 0.19  7.9 ± 1.8% | 8 |
| bucindolol | -10.14 | 1.19  19.9% | -10.14 ± 0.03 | 0.79 ± 0.06  34.4 ± 5.6% | 7 | -10.13 ± 0.03 | 0.86 ± 0.11  26.4 ± 3.8% | 7 | -10.23 ± 0.06 | 0.62 ± 0.12  22.2 ± 7.2% | 6 | -10.06 ± 0.05 | 0.57 ± 0.13  23.9 ± 8.8 | 7 |
| levobunolol | -10.09 | 2.32  59.1% | -10.11 ± 0.03 | 2.48 ± 0.09  67.9 ± 3.9% | 6 | -10.02 ± 0.04 | 2.16 ± 0.11  72.6 ± 3.6% | 6 | -10.03 ± 0.06 | 2.02 ± 0.05  60.9 ± 7.4% | 6 | -9.94 ± 0.04 | 2.01 ± 0.07  71.1 ± 6.6% | 5 |
| timolol | -10.02 | 2.73  49.9% | -9.89 ± 0.04 | 2.61 ± 0.13  58.3 ± 4.4% | 6 | -9.94 ± 0.05 | 60.7 ± 3.5  2.62 ± 0.12 | 6 | -9.96 ± 0.02 | 57.9 ± 3.1  2.38 ± 0.12 | 8 | -9.87 ± 0.06 | 61.0 ± 3.1  2.37 ± 0.19 | 6 |
| carvedilol | -9.94 | 0.11 | -9.94 ± 0.08 | 0.09 ± 0.07 | 9 | -10.04 ± 0.08 | 0.12 ± 0.05 | 9 | -9.90 ± 0.08 | -0.07 ± 0.08 | 11 | -9.93 ± 0.10 | 0.31 ± 0.11 | 9 |
| bupranolol | -9.92 | 1.86 | -9.94 ± 0.05 | 2.08 ± 0.10 | 13 | -9.88 ± 0.03 | 1.96 ± 0.11 | 13 | -9.97 ± 0.04 | 1.74 ± 0.09 | 12 | -9.00 ± 0.04** | 1.93 ± 0.10 | 12 |
| ICI118551 | -9.37 | 1.21 | -9.42 ± 0.04 | 1.59 ± 0.11 | 12 | -9.45 ± 0.03 | 1.53 ± 0.12 | 12 | -9.38 ± 0.05 | 1.15 ± 0.10 | 10 | -8.49 ± 0.04** | 1.47 ± 0.09 | 11 |
| propranolol | -9.27 | 1.67 | -9.38 ± 0.04 | 1.81 ± 0.10 | 12 | -9.34 ± 0.05 | 1.77 ± 0.10 | 12 | -9.32 ± 0.05 | 1.38 ± 0.09 | 11 | -9.14 ± 0.04 | 1.55 ± 0.10 | 11 |
| alprenolol | -9.08 | 2.03 | -9.28 ± 0.05 | 2.09 ± 0.14 | 8 | -9.26 ± 0.02 | 1.90 ± 0.12 | 8 | -9.38 ± 0.06 | 1.76 ± 0.12 | 7 | -9.02 ± 0.03 | 1.61 ± 0.09 | 8 |
| BAAM | -8.74 | 0.91  30.2% | -8.96 ± 0.13 | 0.91 ± 0.05  36.7 ± 2.7% | 6 | -8.85 ± 0.12 | 0.76 ± 0.07  45.4 ± 5.2% | 6 | -8.86 ± 0.08 | 0.70 ± 0.11  32.8 ± 9.3% | 5 | -8.69 ± 0.12 | 0.68 ± 0.06  31.6 ± 4.2% | 6 |
| nadolol | -8.52 | 2.68 | -8.69 ± 0.05 | 2.90 ± 0.10 | 6 | -8.58 ± 0.04 | 2.83 ± 0.07 | 6 | -8.57 ± 0.05 | 2.52 ± 0.09 | 8 | -8.43 ± 0.05 | 2.56 ± 0.10 | 6 |
| nebivolol | -7.44 | 0.47 | -7.59 ± 0.05 | 0.76 ± 0.05 | 6 | -7.60 ± 0.03 | 0.78 ± 0.11 | 6 | -7.30 ± 0.14 | 0.26 ± 0.05 | 8 | -7.50 ± 0.12 | 0.70 ± 0.09 | 6 |

Table S2b – β1-polymorphic variants

|  | β1 wildtype | | CHO-β1-gly49 | | | CHO-β1-arg389 | | |
| --- | --- | --- | --- | --- | --- | --- | --- | --- |
|  | Log K_D_ | Log shift | Log K_D_ | Log shift | n | Log K_D_ | Log shift | n |
| cyanopindolol | -10.14 | 1.26  9.0% | -9.84 ± 0.04 | 1.17 ± 0.11  6.9 ± 0.8% | 7 | -9.83 ± 0.03 | 0.94 ± 0.11  15.4 ± 0.9% | 7 |
| carazolol | -9.68 | 1.36  26.8 | -9.74 ± 0.03 | 1.56 ± 0.09  28.5 ± 2.2% | 6 | -9.74 ± 0.03 | 1.67 ± 0.09  29.2 ± 2.3% | 6 |
| carpindolol | -9.00 | 2.16  71.2% | -8.89 ± 0.02 | 1.70 ± 0.08  76.6 ± 2.8% | 6 | -8.88 ± 0.05 | 1.74 ± 0.09  67.7 ± 3.0% | 6 |
| bucindolol | -9.44 | 1.06  39.1% | -9.26 ± 0.07 | 0.82 ± 0.03  21.3 ± 4.9% | 7 | -9.19 ± 0.08 | 0.78 ± 0.10  31.7 ± 4.9% | 7 |
| levobunolol | -8.37 | 2.62 | -8.35 ± 0.04 | 2.57 ± 0.05 | 6 | -8.42 ± 0.11 | 2.47 ± 0.15 | 6 |
| timolol | -8.73 | 2.89  74% | -8.59 ± 0.02 | 3.31 ± 0.04  81.7 ± 1.5% | 6 | -8.55 ± 0.03 | 3.27 ± 0.11  78.3 ± 2.8% | 6 |
| carvedilol | -9.19 | 0.33 | -9.11 ± 0.06 | 0.37 ± 0.07 | 6 | -9.09 ± 0.04 | 0.22 ± 0.11 | 6 |
| bupranolol | -8.78 | 1.97 | -8.75 ± 0.02 | 2.19 ± 0.06 | 6 | -8.71 ± 0.01 | 2.28 ± 0.06 | 6 |
| ICI 118551 | -6.82 | 1.81 | -6.73 ± 0.02 | 2.00 ± 0.05 | 6 | -6.69 ± 0.02 | 2.10 ± 0.18 | 6 |
| propranolol | -8.32 | 1.68 | -8.20 ± 0.05 | 1.94 ± 0.8 | 6 | -8.16 ± 0.03 | 2.03 ± 0.10 | 6 |
| alprenolol | -8.06 | 2.31 | -8.02 ± 0.03 | 2.03 ± 0.08 | 6 | -8.00 ± 0.02 | 2.00 ± 0.02 | 6 |
| BAAM | -7.99 | 1.17  45.4% | -7.93 ± 0.09 | 1.01 ± 0.04  42.2 ± 4.7% | 7 | -7.88 ± 0.08 | 1.02 ± 0.08  47.0 ± 2.5% | 7 |
| nadolol | -7.54 | 2.83 | -7.39 ± 0.03 | 2.97 ± 0.08 | 6 | -7.27 ± 0.01 | 3.04 ± 0.13 | 6 |
| nebivolol | -8.63 | 0.28 | -8.60 ± 0.08 | 0.28 ± 0.05 | 6 | -8.63 ± 0.07 | 0.34 ± 0.07 | 6 |

**p<0.000001 One-way ANOVA with post hoc Newman-Keuls comparing log K_D_ values obtained in all polymorphic variants with those obtained from the β2-WT. Thus e.g. the log K_D_ for bupranolol and ICI118551 in β2-ile164 is different from that obtained from the β2-WT and all other β2-polymorphisms with p<0.000001. *p<0.000001 One-way ANOVA with post hoc Newman-Keuls comparing log K_D_ values obtained in all polymorphic variants with those obtained from the β2-WT.

Although statistically significantly different from β2-WT alone, carazolol has only 3-fold higher affinity than β2-WT.

Supplementary information Table S3a and S3b

Log EC_50_ and % response compared to that of 10μM isoprenaline from CRE-SPAP production in cell lines stably expressing the naturally occurring polymorphic β-AR variants (CHO-β2-gly16, CHO-β2-gln27, CHO-β2-met34, CHO-β2-ile164, CHO-β1-gly49 and CHO-β1-arg389). For certain compounds a biphasic response was seen in the β1-variants. Here, log EC_50_ at component 1 (EC_50_1) and component 2 (EC_50_2) are given with the % of the response occurring via component 1, and % isoprenaline for the whole response (as in Table 3 in the main manuscript). Values are mean ± sem of n separate experiments. Compounds are arranged in order of β2-efficacy (size of response, as for Table 3).

Table 3a – β2 polymorphic variants

|  | β2-wildtype | | CHO-β2-gly16 | | | CHO-β2-gln27 | | | CHO-β2-met34 | | | CHO-β2-ile164 | | |
| --- | --- | --- | --- | --- | --- | --- | --- | --- | --- | --- | --- | --- | --- | --- |
|  | Log EC_50_ | % isop | Log EC_50_ | % isop | n | Log EC_50_ | % isop | n | Log EC_50_ | % isop | n | Log EC_50_ | % isop | n |
| bucindolol | -9.75 | 66.6 | -9.45 ± 0.09 | 35.3 ± 3.5 | 11 | -9.38 ± 0.05 | 29.5 ± 3.0 | 11 | -8.87 ± 0.35 | 7.7 ± 2.3 | 11 | -9.63 ± 0.24 | 9.35 ± 1.2 | 11 |
| cyanopindolol | -9.94 | 44.6 | -9.50 ± 0.18 | 23.2 ± 2.4 | 9 | -9.82 ± 0.22 | 14.0 ± 2.0 | 9 | No response |  | 8 | -9.79 ± 0.16 | 5.8 ± 1.3 | 8 |
| alprenolol | -9.59 | 41.3 | -9.39 ± 0.13 | 22.3 ± 3.5 | 8 | -9.23 ± 0.06 | 19.6 ± 3.4 | 9 | No response |  | 9 | -9.01 ± 0.23 | 6.6 ± 1.1 | 8 |
| BAAM | -8.81 | 27.2 | -8.83 ± 0.21 | 14.0 ± 3.1 | 8 | -8.66 ± 0.24 | 5.8 ± 0.9 | 8 | No response |  | 8 | No response |  | 9 |
| carpindolol | -9.64 | 23.4 | -9.34 ± 0.16 | 17.6 ± 2.7 | 8 | -9.40 ± 0.27 | 7.2 ± 1.7 | 8 | No response |  | 8 | No response |  | 8 |
| carvedilol | -9.35 | 18.5 | -10.03 ± 0.31 | 11.4 ± 2.6 | 11 | -9.18 ± 0.43 | 3.8 ± 1.6 | 12 | No response |  | 11 | No response |  | 12 |
| carazolol | -10.14 | 9.7 | -9.55 ± 0.47 | 3.97 ± 1.3 | 9 | No response |  | 8 | No response |  | 8 | No response |  | 8 |
| propranolol | -9.66 | 9.2 | No response |  | 8 | No response |  | 9 | No response |  | 9 | No response |  | 9 |
| bupranolol | No response |  | No response |  | 5 | No response |  | 5 | No response |  | 5 | No response |  | 5 |
| ICI118551 | No response |  | No response |  | 5 | No response |  | 5 | No response |  | 5 | No response |  | 5 |
| levobunolol | No response |  | No response |  | 5 | No response |  | 5 | No response |  | 5 | No response |  | 5 |
| nadolol | No response |  | No response |  | 5 | No response |  | 5 | No response |  | 5 | No response |  | 5 |
| nebivolol | No response |  | No response |  | 5 | No response |  | 5 | No response |  | 5 | No response |  | 5 |
| timolol | No response |  | No response |  | 5 | No response |  | 5 | No response |  | 5 | No response |  | 5 |

Table 3b – β1 polymorphic variants

|  | β1-wildtype | | | | CHO-β1-gly49 | | | | | CHO-β1-arg389 | | | | |
| --- | --- | --- | --- | --- | --- | --- | --- | --- | --- | --- | --- | --- | --- | --- |
|  | Log EC_50_1 | Log EC_50_2 | %site 1 | % isop | Log EC_50_1 | Log EC_50_2 | %site 1 | % isop | n | Log EC_50_1 | Log EC_50_2 | %site 1 | % isop | n |
| bucindolol | -9.08 |  |  | 78.5 | -8.26 ± 0.10 |  |  | 43.9 ± 2.6 | 11 | -8.79 ± 0.07 |  |  | 69.6 ± 4.6 | 11 |
| cyanopindolol | -8.80 |  |  | 73.2 | -7.47 ± 0.24 |  |  | 36.5 ± 5.1 | 9 | -8.53 ± 0.11 |  |  | 71.7 ± 7.6 | 9 |
| alprenolol | -8.54 | -6.22 | 51.8 | 65.3 | -7.50 ± 0.17 |  |  | 16.9 ± 3.4 | 7 | -8.34 ± 0.18 | -5.95 ± 0.26 | 58.7 ± 4.9 | 63.6 ± 6.3 | 9 |
| BAAM | -7.82 |  |  | 67.7 | -7.24 ± 0.23 |  |  | 14.0 ± 3.4 | 9 | -7.64 ± 0.05 |  |  | 54.8 ± 6.3 | 9 |
| carpindolol | -9.06 | -6.85 | 51.2 | 82.9 | -9.00 ± 0.29 | -6.27 ± 0.18 | 36.5 ± 3.2 | 44.3 ± 2.8 | 8 | -8.57 ± 0.16 | -6.35 ± 0.29 | 63.6 ± 6.5 | 94.8 ± 6.0 | 8 |
| carvedilol | -7.93 |  |  | 33.3 | -8.42 ± 0.29 |  |  | 8.8 ± 2.0 | 12 | -8.12 ± 0.11 |  |  | 29.8 ± 2.5 | 11 |
| carazolol | -7.89 |  |  | 58.7 | -7.20 ± 0.21 |  |  | 16.0 ± 3.7 | 8 | -7.85 ± 0.10 |  |  | 53.4 ± 5.9 | 8 |
| propranolol | -6.88 |  |  | 15.0 | No response |  |  |  | 9 | -6.90 ± 0.20 |  |  | 21.6 ± 3.7 | 9 |
| bupranolol | No response |  |  |  | No response |  |  |  | 5 | No response |  |  |  | 5 |
| ICI118551 | No response |  |  |  | No response |  |  |  | 5 | No response |  |  |  | 5 |
| levobunolol | No response |  |  |  | No response |  |  |  | 5 | No response |  |  |  | 5 |
| nadolol | No response |  |  |  | No response |  |  |  | 5 | No response |  |  |  | 5 |
| nebivolol | No response |  |  |  | No response |  |  |  | 5 | No response |  |  |  | 5 |
| timolol | No response |  |  |  | No response |  |  |  | 5 | No response |  |  |  | 5 |

Supplementary Table S4

Summary of the K_D_ values for ^3^H-CGP12177 and receptor expression levels for the cell lines used in the manuscript

|  | K_D_ for ^3^H-CGP1277 | Receptor expression level | reference |
| --- | --- | --- | --- |
| CHO-β2 | 0.22nM | 514 fmol/mg protein | Determined in this manuscript, also see reference 63 |
| CHO-β2-gly16 | 0.14nM | 340 fmol/mg protein | Reference 61 |
| CHO-β2-gln27 | 0.14nM | 368 fmol/mg protein | Reference 61 |
| CHO-β2-met34 | 0.13nM | 134 fmol/mg protein | Reference 61 |
| CHO-β2-ile164 | 0.13nM | 152 fmol/mg protein | Reference 61 |
| CHO-β1 | 0.42nM | 1133 fmol/mg protein | Determined in this manuscript, also see reference 63 |
| CHO-β1-gly49 | 0.38nM | 819 fmol/mg protein | Reference 62 |
| CHO-β1-arg389 | 0.45nM | 1068 fmol/mg protein | Reference 62 |
